# Risk behaviors and reasons for not getting tested for HIV among men who have sex with men in Peru

**DOI:** 10.1186/1742-4690-9-S1-P111

**Published:** 2012-05-25

**Authors:** Magaly M Blas, Isaac E Alva, Robinson Cabello, Cesar Carcamo, Ann E Kurth

**Affiliations:** 1 Cayetano Heredia Peruvian University, Lima, Peru

## Introduction

Men who have sex with men (MSM) account for the greatest burden of the HIV epidemic in Peru. Given that MSM are frequent users of the Internet, understanding the risk behaviors and the reasons for not getting tested among MSM who surf the Internet may improve the tailoring of future online behavioral interventions.

## Methods

From October 2007 to April 2008, we conducted an online survey among users of seven Peruvian gay websites.

## Results

We received 1,481 surveys, 1,301 of which were included in the analysis. The median age of the participants was 22.5 years (range 12-71), 67% were homosexual, and the remainder was bisexual. Of survey respondents, 49.4% had never been tested for HIV and only 11.3% were contacted in-person during the last year by peer health educators from the Peruvian Ministry of Health and NGOs. Additionally, 50.8% had unprotected anal or vaginal sex at last intercourse, and a significant percentage reported a condom broken (22.1%), slipped (16.4%) or sexual intercourse initiated without wearing a condom (39.1%). The most common reasons for not getting tested for HIV among high-risk MSM were "I fear the consequences of a positive test result" (n = 55, 34.4%), and "I don't know where I can get tested" (n = 50, 31.3%).

## Conclusions

A small percentage of Peruvian MSM who answered our online survey, were reached by traditional peer-based education programs. Given that among high-risk MSM, fear of a positive test result and lack of awareness of places where to get tested are the most important reasons for not taking an HIV test, Internet interventions aimed at motivating HIV testing should work to reduce fear of testing and increase awareness of places that offer free HIV testing services to MSM.

